# An innate interaction between IL-18 and the propeptide that inactivates its precursor form

**DOI:** 10.1038/s41598-019-42661-5

**Published:** 2019-04-16

**Authors:** Naotaka Tsutsumi, Ayumi Yokota, Takeshi Kimura, Zenichiro Kato, Toshiyuki Fukao, Masahiro Shirakawa, Hidenori Ohnishi, Hidehito Tochio

**Affiliations:** 1 Department of Biophysics, Graduate School of Science, Kyoto University, Sakyo-ku, Kyoto, 606-8502 Japan; 2 Department of Molecular Engineering, Graduate School of Engineering, Kyoto University, Katsura, Nishikyo-ku, Kyoto, 615-8510 Japan; 3 Institute for Integrated Cell-Material Sciences, Kyoto University, Sakyo-ku, Kyoto, 606-8501 Japan; 4 Department of Pediatrics, Graduate School of Medicine, Gifu University, Yanagido 1-1, Gifu, 501-1194 Japan; 5 Structural Medicine, Medical Information Sciences Division, The United Graduate School of Drug Discovery and Medical Information Sciences, Gifu University, Gifu, 501-1194 Japan

## Abstract

Uncontrolled secretion of mature interleukin (IL)-1β and IL-18 is responsible for severe autoinflammatory or autoimmune disorders and various allergic diseases. Here we report an intramolecular interaction between IL-18 and its propeptide, which is proteolytically removed from its precursor proIL-18 during maturation. The intramolecular interaction was recapitulated intermolecularly using recombinant propeptide. These results suggest the possibility of developing a novel class of peptide-based IL-18 inhibitors that could serve as therapeutic agents for IL-18-related inflammatory diseases.

## Introduction

Interleukin (IL)-18 is a proinflammatory cytokine belonging to the IL-1 family (IL-1F) and potently stimulates interferon (IFN)-γ production to protect hosts against infections^1,2^. All IL-1F ligand agonists, including IL-18, are synthesized in precursor forms (proIL-1F)^3^ bearing N-terminal propeptide sequences (PPs) with lengths varying from 4 to 116 amino acids. The most of proIL-1Fs are either completely inactive or less active, and the maturation step, in which PP is removed via enzymatic cleavage, is necessary for full activation. For instance, inflammatory IL-18 and IL-1β are produced as precursors called proIL-18 and proIL-1β, respectively. The precursors remain inactive in the cytosol until a signal, inflammasome activation, induces maturation. The inflammasome is a multi-protein assembly composed of three proteins, a nucleotide-binding oligomerization domain (NOD)-like receptor, apoptosis-associated speck-like protein containing a CARD (ASC) and caspase-1, and is formed upon detection of pathogens or other harmful substances, such as reactive oxygen species and urate crystals, in the cytosol. The assembly then autocatalyzes activation of the cysteine protease caspase-1^4^, which removes PPs from the precursors to produce IL-1β and IL-18. These mature forms are then secreted extracellularly to exert their biological functions; however, the mechanism underlying this secretion remains controversial^5–7^. Secreted IL-1β and IL-18 bind to their receptor pairs (IL-1RI/IL-1RAcP^8^ and IL-18Rα/IL-18Rβ^9^, respectively) on target cells to initiate intracellular MyD88-dependent signaling. This ultimately activates NF-κB, which upregulates expression of various inflammatory cytokines. In a sense, IL-1β and IL-18 PPs securely lock in the cytokines’ powerful proinflammatory activity so that they cannot readily trigger severe inflammatory diseases^10–13^. In fact, proIL-1β and proIL-18 are exposed to the extracellular space upon pyroptotic cell death; however, maturation is still required for them to engage their receptors, eliciting intracellular signaling and the inflammatory response^14–16^. Especially in the case of IL-18, the fast process of enzymatic maturation of the stored precursor, rather than the slower production of mature proteins by transcription and translation, may facilitate rapid release of active cytokine in response to invading pathogens. In contrast, other IL-1F members, such as proIL-1α and proIL-33, are somewhat active even when their PPs remain intact. They can activate their receptors on target cells when released from dying cells although the activity of proIL-33 is weaker than that of its mature form^14,17^.

Recent studies have shown that inflammasomes play a crucial role in regulating IL-1β and IL-18 secretion, and many genetic disorders in their components are responsible for high circulating levels of these cytokines, resulting in autoinflammatory syndromes. The most typical case is cryopyrin-associated periodic syndrome (CAPS), in which specific mutations in the NOD-like receptor NACHT, LRR, and PYD domains-containing protein 3 (NLRP3)^10^ induce uncontrolled NLRP3 inflammasome activation, which eventually leads to chronic inflammation because of IL-1β hyper-production. Similarly, certain mutations in the NLR family CARD domain-containing protein 4 (NLRC4) evoke periodic fevers of lethal macrophage-activating syndrome (MAS)^11–13^, in which patients suffer from extremely high blood levels of IL-18 in addition to elevated levels of IL-1β. Interestingly, IL-18 levels in NLRC4-MAS patients remain high for a long period, even after IL-1β blockade. These findings reinforce the concept that PPs as important regulators of proinflammatory IL-1β and IL-18.

In addition to NLRC4-MAS, IL-18 is associated with various severe chronic inflammatory diseases such as CAPS, familial Mediterranean fever, adult-onset Still’s disease (AOSD), pyogenic arthritis, pyoderma gangrenosum, acne syndrome, and systemic juvenile idiopathic arthritis with MAS^18–22^. High blood concentrations of IL-18 are also found in patients with allergic diseases, including bronchial asthma, atopic dermatitis, inflammatory bowel disease, and the X-linked inhibitor of apoptosis (XIAP) deficiency^23–26^. Genetic polymorphisms have also been reported in the IL-18 or IL-18 receptor genes of patients with autoimmune diseases, allergic reactions, and neurological/metabolic syndromes^27–31^.

Neutralizing IL-1β by using either anti-IL-1β antibodies (Canakinumab) or engineered soluble receptors (Rilonacept), or by antagonizing the receptor with IL-1Ra (Anakinra), treat IL-1β-related autoinflammatory diseases effectively^32–35^. Similarly, anti-IL-18 antibodies inhibit the development of atopic dermatitis and asthma-like phenotypes in mouse models^36,37^. A recombinant human IL-18-binding protein (IL-18BP, Tadekinig Alfa), an endogenous antagonist of IL-18, is also in mid-late phase clinical trials for AOSD, XIAP deficiency and NLRC4-MAS^38–40^.

Here, we demonstrate an intramolecular interaction between the mature region of IL-18 and its PP, providing a solid framework for further investigations of PPs’ roles in IL-1F ligands’ molecular function. These results also suggest the possibility of developing a new class of peptide-based drugs to treat IL-1F-associated inflammatory diseases.

## Methods

### Construction of expression vectors

The coding region of the full-length human IL-18 precursor (proIL-18, NM_001562.3, residues 1–193) was amplified from a human cDNA library by a polymerase chain reaction and cloned into a pET-28a vector (Novagen, WI, USA), which was engineered to contain a 6× His-tagged N-terminal small ubiquitin-like modifier (SUMO)-3 (NM_006936.2, residues 14–92) for protein expression and purification. The plasmids for mature IL-18 (residues 37–193) and proIL-18PP (residues 1–36) were derived from the proIL-18 construct by deleting residues 1–36, or by replacing Y37 with a stop codon, respectively, using a KOD -Plus- Mutagenesis kit (Toyobo, Osaka, Japan). The constructs for the N-terminal-truncated proIL-18 variants (proIL-18ΔNs), namely, proIL-18Δ8N (residues 9–193), proIL-18Δ10N (residues 11–193), proIL-18Δ12N (residues 13–193), proIL-18Δ13N (residues 14–193) and proIL-18Δ22N (residues 23–193), were prepared in the same manner as the IL-18 expression plasmid.

### Protein expression and purification

The expression vectors for proIL-18, proIL-18ΔNs, IL-18 and proIL-18PP were transformed into *E. coli* BL21(DE3) cells, and the bacteria were grown in LB medium or M9 minimal media containing either ^15^NH_4_Cl/^12^C_6_H_12_O_6_ or ^15^NH_4_Cl/^13^C_6_H_12_O_6_ at 37 °C in the presence of 25 μg/mL kanamycin. Cultures were cooled on ice to 18 °C when the optical density at 600 nm reached 1.0, and protein expression was induced by adding 1.0 mM IPTG. Cells were harvested after incubating for 16 h at 18 °C, resuspended at 4 °C in lysis buffer (20 mM Tris.HCl, pH 8.0, 150 mM NaCl, 20 mM imidazole and 0.5–2 mM DTT), and supplemented with 1 mM PMSF for proIL-18, proIL-18ΔNs and IL-18 or with cOmplete EDTA-free Protease Inhibitor Cocktail (Roche Applied Science, Penzberg, Germany) for proIL-18PP. After sonication on ice and centrifugation, the supernatant was applied to an Ni affinity column (cOmplete His-Tag Purification Resin, Roche Applied Science) equilibrated with lysis buffer. After washing with lysis buffer, followed by detergent buffer (20 mM Tris.HCl, pH 8.0, 150 mM NaCl, 20 mM imidazole, 1% Triton X-100 and 2 mM DTT), high-salt buffer (20 mM Tris.HCl, pH 8.0, 1 M NaCl, 20 mM imidazole and 2 mM DTT) and then again with lysis buffer, the His-SUMO tag was removed by digestion with a homemade SUMO-specific protease GST-SENP2 for 16 h at 4 °C on column. The protein solution was eluted and allowed to flow through a glutathione sepharose (GE Healthcare, Little Chalfont, UK) to remove the protease. The flow through was further purified using a HiTrap Q anion exchange column (GE Healthcare) and eluted with an NaCl gradient from 50 mM to 500 mM. This was followed by a size-exclusion chromatography on a HiLoad 16/60 Superdex 75 column (GE Healthcare). The gel-filtration column was equilibrated with either NMR buffer (20 mM potassium phosphate, pH 6.0, 150 mM KCl and 1 mM TCEP) for proIL-18, proIL-18ΔNs and IL-18 or with 300 mM ammonium acetate for proIL-18PP. The proIL-18PP eluate was quantitated, aliquoted and lyophilized.

### NMR spectroscopy

NMR spectra were measured on Bruker Avance II 700 MHz, Avance 600 MHz and Avance III 500 MHz spectrometers equipped with cryogenic probes. The samples for NMR analyses were dissolved in either NMR buffer or caspase-1 reaction buffer (20 mM HEPES-Na pH 7.4, 100 mM NaCl, 1 mM EDTA and 10 mM DTT), which was exchanged by dialysis, and 5 v/v % D_2_O was added before measurements. To detect interactions between IL-18 and proIL-18PP, 28.4 nmol of lyophilized [^15^N]-proIL-18PP was directly dissolved in 300 μL of either NMR buffer or 320 μM non-labeled IL-18 in NMR buffer. Reverse detection was performed with 240 μL of 50 μM [^15^N]-IL-18 in the presence and absence of approximately 5 molar equivalents (eq.) of proIL-18PP in caspase-1 reaction buffer. Chemical shift assignments for free proIL-18PP were based on a HNCACB/CBCA(CO)NH data set. To record ^1^H-^15^N SOFAST HMQC spectra with *in situ* caspase-1 cleavage, 10 units of caspase-1 (Enzo Life Sciences, NY, USA) was added to 300 μL of 200 μM proIL-18. Each 2D spectrum was collected for approximately 71 min, and 20 spectra were recorded over approximately 24 hours after adding caspase-1. For comparison, a reaction mixture was prepared, using the same lot of proteins and under the same conditions as the *in situ* NMR experiment, and was analyzed by SDS-PAGE at various time points to confirm the enzymatic activities. To compare the thermotolerance of IL-18 and proIL-18, ^1^H-^15^N SOFAST HMQC spectra were sequentially recorded at 40 °C, 45 °C and 50 °C. The spectra were processed using NMRPipe^41^ or Bruker TopSpin version 3.5pl7 and analyzed using Sparky^42^ and CcpNmr Analysis^43^ version 2.4.2.

### Circular dichroism (CD) spectroscopy

CD spectra were collected on a J-700 spectrophotometer (JASCO, MD, USA) from 250 to 200 nm at 0.1 nm intervals. The proIL-18 and IL-18 concentrations were adjusted to 0.2 mg/mL in NMR buffer and data were reported as molar ellipticity θ (deg cm^2^/dmol). For thermal studies, the molar ellipticity at 215 nm were recorded from 20 °C to 80 °C at a heating rate of 1 °C/min, with 0.2 °C intervals. Data were processed using Igor Pro 6.2.2.0 (WaveMetrics, OR, USA) using moving averages of 1 nm and 1 °C for the comparison of spectral pattern and thermal scanning, respectively.

## Results

### Detection of the interaction between IL-18 and proIL-18PP

**Figure 1:**
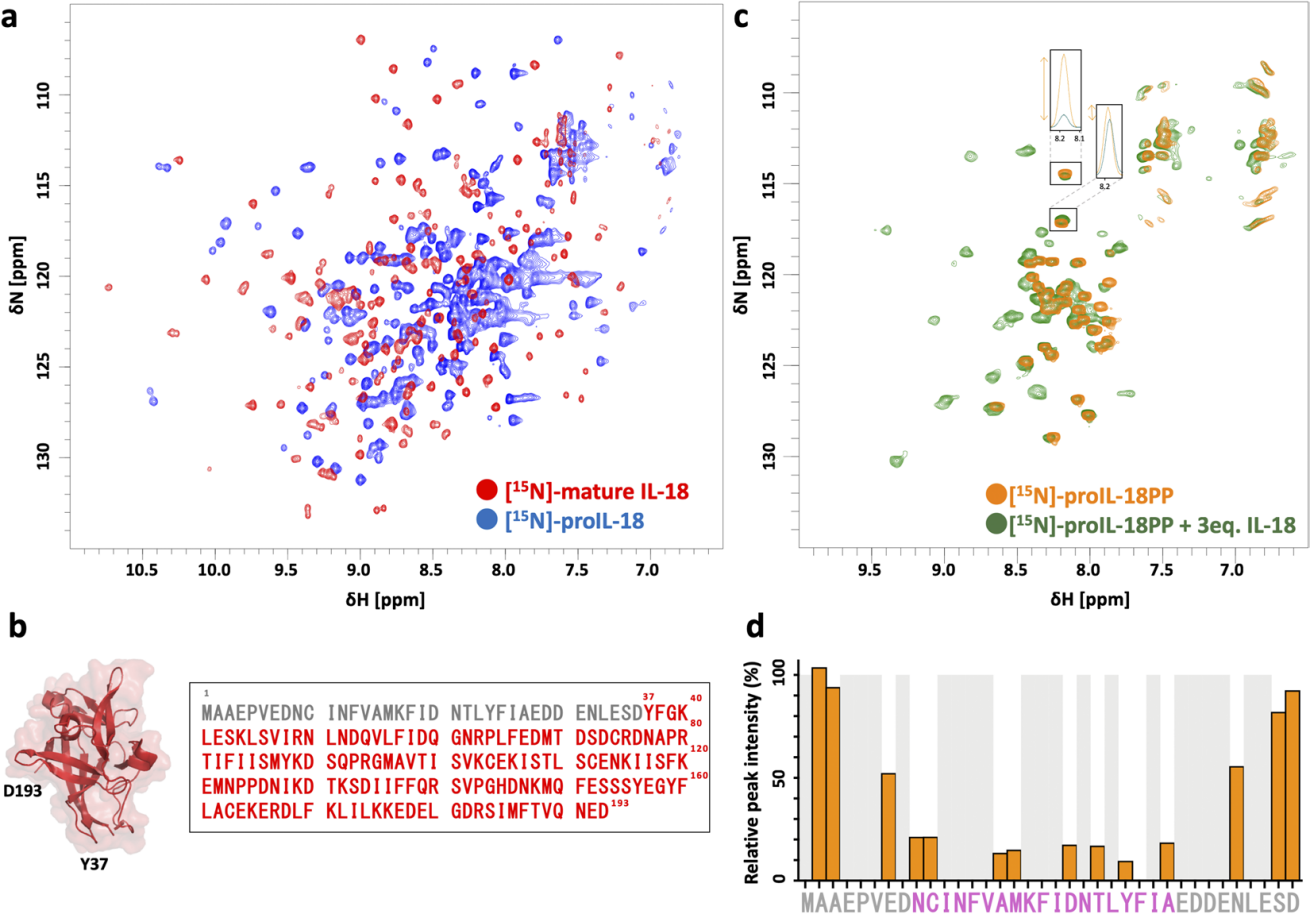
(**a**) Comparison of the 2D ^1^H-^15^N SOFAST HMQC spectra of IL-18 (red) and proIL-18 (blue) showed a drastic difference in spectral patterns. The spectra were measured on a Bruker Avance II 700 MHz at 30 °C in NMR buffer. (**b**) Molecular structure of IL-18’s mature region (PDB ID: 3WO4) and full-length proIL-18’s amino acid sequence. Gray and red letters indicate residues in IL-18’s propeptide and mature regions, respectively. (**c**) Changes in the ^1^H-^15^N NMR spectra of [^15^N]-proIL-18PP (orange) upon binding to IL-18 (green). The spectra were measured on a Bruker Avance 600 MHz at 25 °C. Either buffer or three molar equivalents of non-labeled IL-18 in buffer were added to lyophilized [^15^N]-proIL-18PP to prepare the samples. Two representative peaks are displayed as 1D ^1^H projections. The ^1^H chemical shift distribution for free proIL-18PP amides was narrow, between 8.4 and 8.0 ppm, whereas the mixture exhibited a much wider distribution. (**d**) Relative intensities of cross-peaks of free [^15^N]-proIL-18PP in the absence and presence of three eq. IL-18. The cross-peak intensities decreased remarkably in the presence of IL-18 for amino acid residues from Asn9 to Ala27 (pink). Reliable peak intensities were not obtained for residues with gray backgrounds because of peak overlap.

We first prepared mature IL-18 and proIL-18, the precursor of IL-18, for analysis by NMR spectroscopy. All recombinant IL-18 variants were expressed with an N-terminal SUMO tag to precisely produce the polypeptide sequences of interest; an analogous method has been reported to produce fully bioactive IL-18^44^. The 2D ^1^H-^15^N HMQC spectrum of [^15^N]-IL-18 prepared in this way was essentially identical to the one we obtained in our previous structural studies of IL-18^9,45^, indicating that the sample protein had the proper tertiary structure to form the signaling ternary complex. ProIL-18 exhibited a monomeric, mono-disperse nature upon size-exclusion chromatography, as did IL-18 (Supplementary Fig. 1a and ref.^46^). However, the 2D ^1^H-^15^N HMQC spectrum of [^15^N]-proIL-18 was dramatically different from [^15^N]-IL-18 (Fig. 1a and ref.^9^), suggesting that the PP (Fig. 1b right, residues 1–36) makes extensive contact with the mature region (Fig. 1b right, residues 37–193) and/or prevents the proper folding of the mature region via certain intramolecular interactions. The cross-peaks were substantially broadened, likely reflecting relatively weak interactions and low structural homogeneity of the molecule. In terms of function, the distinct structural state of proIL-18, revealed by the NMR spectra, is consistent with its inability to activate IL-18 receptors, triggering IL-18 signaling^47,48^. Since the receptors recognize large surface areas of IL-18^9^, the clearly distinct structure of proIL-18 should hamper binding.

To determine whether the observed intramolecular interaction can be reproduced intermolecularly, we next examined the binding between a peptide comprising the PP sequence (proIL-18PP, Supplementary Fig. 1b) and mature IL-18. The structure of recombinant [^15^N]-proIL-18PP was completely disordered since the chemical shift dispersion of the main-chain amide protons in the 2D ^1^H-^15^N HMQC spectrum was narrow and lay in a range of 8.4 to 8.0 ppm^49^ (Fig. 1c and Supplementary Fig. 2a). The secondary structure propensity was evaluated based on the backbone heavy atom assignments of free [^13^C/^15^N]-proIL-18PP (Supplementary Fig. 2b), confirming that there is essentially no secondary structure in the free peptide.

**Figure 2:**
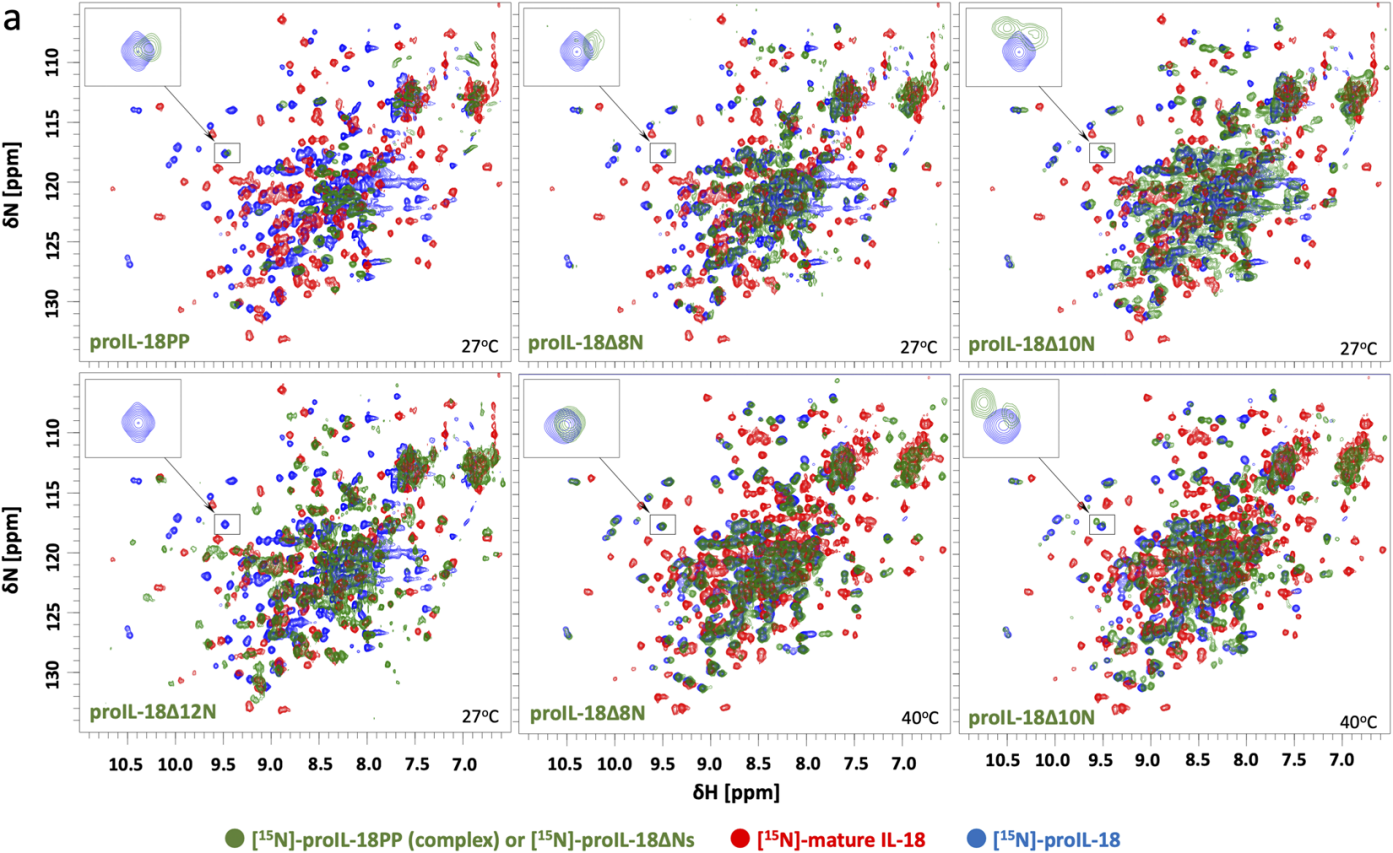
Spectral conservation between proIL-18, IL-18 and proIL-18PP complexed with IL-18 and proIL-18ΔNs. One representative peak shared between proIL-18 and proIL-18PP (complex), marked with a square, is shown in close-up for comparison. [^15^N]-proIL-18Δ8N yielded a very similar spectrum to that of full-length [^15^N]-proIL-18, whereas the spectrum of [^15^N]-proIL-18Δ12N is related to that of mature [^15^N]-IL-18. [^15^N]-proIL-18Δ10N appears to be an intermediate form between precursor and mature IL-18. All proIL-18ΔNs spectra were measured on a Bruker Avance III 500 MHz. ProIL-18 and IL-18 spectra were measured on a Bruker Avance II 700 MHz and Avance 600 MHz, respectively.

However, the NMR spectrum of [^15^N]-proIL-18PP changed substantially in the presence of three molar equivalents of IL-18, with new cross-peaks appearing at a δ(^1^H) of 9.5–8.5 ppm, indicating specific interaction between proIL-18PP and IL-18 and the formation of an ordered structure in the peptide (Fig. 1c). These new peaks can be superimposed on certain peaks of [^15^N]-proIL-18 (Fig. 2a, top left), suggesting that the peptide experiences the same environment as proIL-18. In other words, proIL-18PP’s binding to IL-18, at least partly, recapitulates the native intramolecular interactions within proIL-18. Although we were unable to assign the new cross-peaks, a certain portion of proIL-18PP was thought to form a β-strand structure based on the amide ^1^H chemical shifts of the peaks (Supplementary Fig. 2c). This interpretation agrees with secondary structure prediction, where Cys10-Ile19 and Thr22-Ala27 adopt short β-strand configurations (Supplementary Fig. 2d). Intriguingly, ^1^H-^15^N NMR signals from amino acids residues in the predicted β-strands were substantially broadened during the titration (Fig. 1d).

Based on the ^1^H-^15^N cross-peak intensities of [^15^N]-proIL-18PP in the presence of three molar equivalents of IL-18, the dissociation constant (K_D_) of the interaction was evaluated as >36 μM. This affinity is in the moderate range, however, in full-length proIL-18, these two regions are connected into a single molecule by a peptide bond, so that they should assemble more tightly. As shown in Fig. 1d, when three molar equivalents of IL-18 were present, the cross-peak intensities of free [^15^N]-proIL-18PP decreased, which was substantial for amino acid residues from Asn9 to Ala27. This observation suggests this region that mainly interacts with IL-18, while the remainder of the peptide does not.

To further explore the region in the PP sequence that is essential for IL-18 binding, we next recorded the ^1^H-^15^N HMQC spectra of a series of N-terminally truncated proIL-18 derivatives (proIL-18ΔNs) and compared them to full-length proIL-18 and mature IL-18 (Fig. 2). [^15^N]-proIL-18Δ8N, in which eight N-terminal residues were truncated, produced a very similar spectral pattern to [^15^N]-proIL-18 (Fig. 2 middle) with the signature cross-peak appeared during [^15^N]-proIL-18PP/IL-18 titration (squared peak in Fig. 2). This observation indicates that the first eight residues are dispensable for the interaction between the pro and mature regions of proIL-18. Because the spectral comparison did not reveal any obvious differences in the locations of the well-isolated cross-peaks, the first eight amino acid residues of proIL-18 may adopt a random coil whose cross-peaks are presumably buried in the crowded and indistinguishable region at approximately a δ(^1^H) of 8.4–8.0 ppm. Additional derivatives were also tested; intriguingly, after deleting the first ten residues of proIL-18 (proIL-18Δ10N, Fig. 2 right), the spectra was broadened substantially, presumably because of intermediate states between proIL-18 and IL-18. Furthermore, the reference cross-peak of full-length proIL-18 began to change (squared peak in Fig. 2). In addition, deletion of the first 12 residues (proIL-18Δ12N, Fig. 2 bottom left) completely altered the spectrum to resemble the mature protein, indicating a critical importance of residues near Ile11. Further deletion did not produce major spectral changes, and proIL-18Δ13N and proIL-18Δ22N yielded essentially the same spectra as proIL-18Δ12N (Supplementary Fig. 3). Taken together, these data suggest that residues 9–11 play a central role in the interaction, although residues 12–27 probably also participate in IL-18 binding.

**Figure 3:**
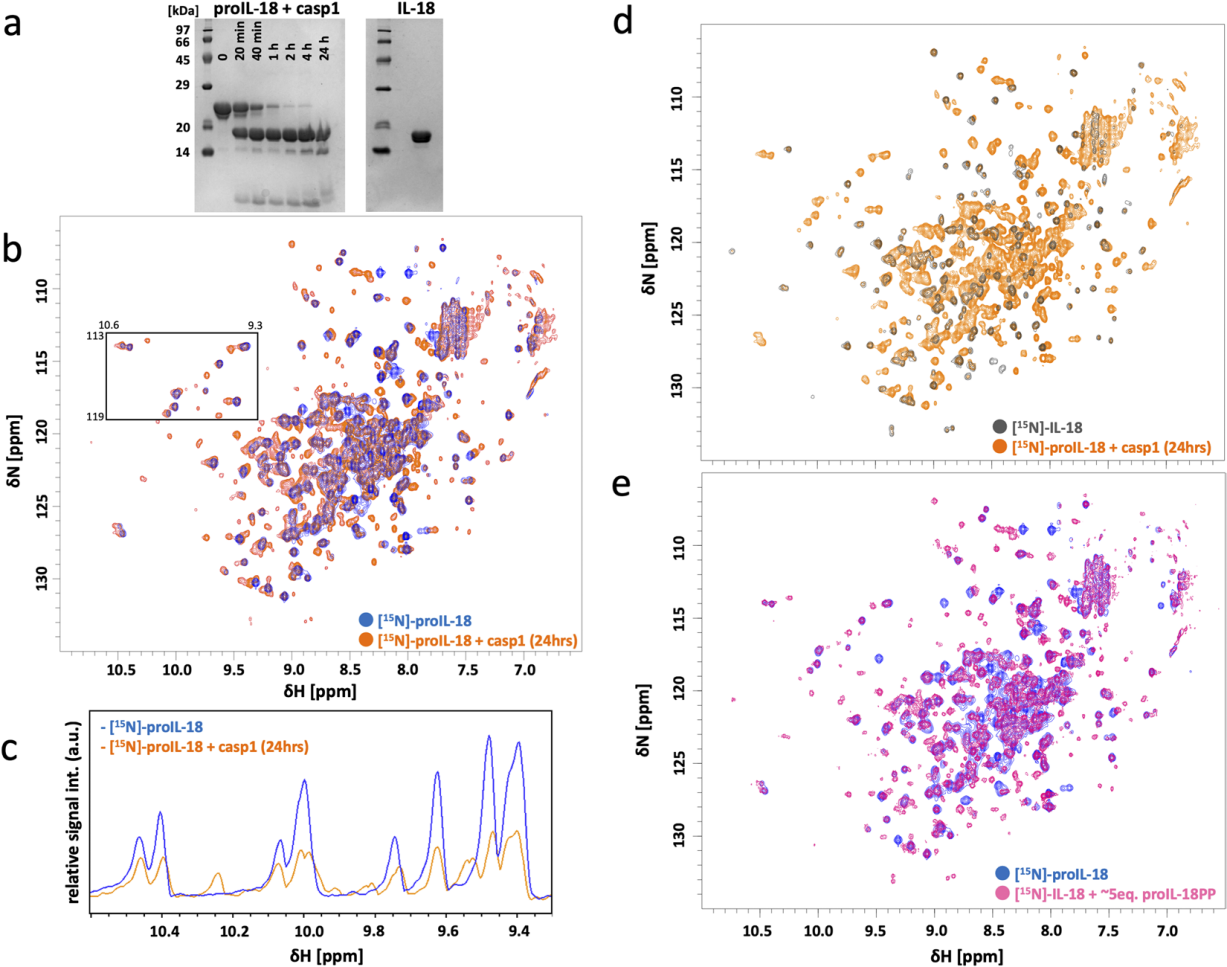
NMR spectra of IL-18 in the presence of proIL-18PP. (**a**–**c**) Caspase-1 reaction of proIL-18 at 37 °C in caspase-1 reaction buffer. (**a**) SDS-PAGE analysis of the reaction at various time points, indicated on the gel. The two images are from two different SDS-PAGE runs (Supplementary Fig. 5). (**b**) ^1^H-^15^N SOFAST HMQC spectra showing [^15^N]-proIL-18 before (blue) and after (orange) caspase-1 digestion. (**c**) ^1^H 1D projection of the square region in (**b**) showing decay of the proIL-18 derived peaks. (**d**) Spectral comparison of [^15^N]-IL-18 (gray) and caspase-1 cleaved [^15^N]-proIL-18 (orange, from **b**). (**e**) Overlaid spectra of [^15^N]-proIL-18 (blue) and [^15^N]-IL-18 in the presence of approximately five molar equivalents of non-labeled proIL-18PP (pink). All ^1^H-^15^N SOFAST HMQC spectra were measured on a Bruker Avance II 700 MHz at 37 °C in caspase-1 reaction buffer.

In cells, proIL-18 maturation occurs via cleavage by caspase-1. To obtain insights into this reaction, we performed *in situ* NMR analysis of the maturation reaction in a test tube, wherein ^1^H-^15^N HMQC spectra of [^15^N]-proIL-18 were sequentially recorded in the presence of active caspase-1 (Fig. 3a–c and Supplementary Fig. 4a). The majority of [^15^N]-proIL-18 was digested within one hour and the reaction was complete after 24 hours, according to SDS-PAGE analysis (Fig. 3a). However, even in the NMR spectrum recorded 24 hours after the addition of caspase-1, essentially all cross-peaks for [^15^N]-proIL-18 remained, although their intensity had decreased by approximately 50% (Fig. 3b,c). In addition, new cross-peaks corresponding to mature IL-18 were apparent, indicating that some fraction of proIL-18 was structurally converted to the mature form (Fig. 3d). These observations indicate that ~50% of the generated proIL-18PP was still in complexed with the mature region, even after cleavage by caspase-1. In fact, considering the initial concentration of proIL-18 in this experiment (200 μM), the K_D_ can be estimated from the NMR spectra (Fig. 3b) as approximately 100 μM, similar to the value determined by titration (Fig. 1c). To confirm the spectral pattern of the mature region of IL-18 in the peptide-bound state, we measured ^1^H-^15^N HMQC spectrum of [^15^N]-IL-18 in the presence of approximately five molar equivalents of non-label proIL-18PP. The yielded spectrum had the similar features to proIL-18, as was in the caspse-1 cleaved experiment but with no signal from free and complexed proIL-18PP (Fig. 3e and Supplementary Fig. 4b).

### ProIL-18 is more stable than mature IL-18

**Figure 4:**
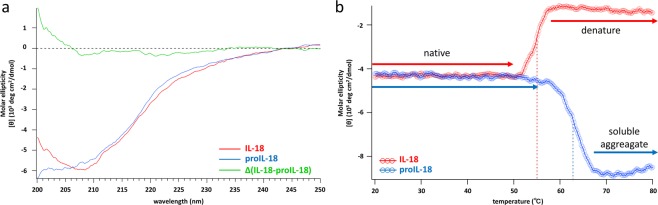
Comparison of CD spectra between IL-18 (red) and proIL-18 (blue) in NMR buffer. (**a**) CD spectra recorded at 20 °C between 200 nm and 250 nm. The difference spectrum is shown in green. (**b**) Temperature dependent changes in CD values at 215 nm from 20 °C to 80 °C.

To compare the secondary structures and folding properties of proIL-18 and IL-18, we collected circular dichroism (CD) spectra in NMR buffer containing 150 mM NaCl (Fig. 4). Consistent with the reported β-trefoil structure^9,45^ with abundant β-hairpins and short connecting loops, IL-18 produced a broad, negative peak at 208 nm (Fig. 4a, red) with a spectral pattern very similar to the reported CD spectra of IL-1Ra and IL-36γ^50,51^. The negative band of proIL-18 was broadened and shifted to a lower wavelength relative to IL-18 (Fig. 4a, blue), indicating a slightly higher random coil content in proIL-18. We then performed thermal scanning by recording CD values at 215 nm^50^ from 20 °C to 80 °C at a heating rate of 1 °C/min (Fig. 4b). IL-18 denatured at a melting temperature (T_m_) of 55 °C, while the CD value of proIL-18 remained unchanged at this point. The CD value of proIL-18 began to drop near 58 °C did so gradually to approximately 68 °C, where it became stable. This decrease in the CD value can be attributed to soluble aggregation^52^ and the midpoint of the transition between the two states was 63 °C.

**Figure 5:**
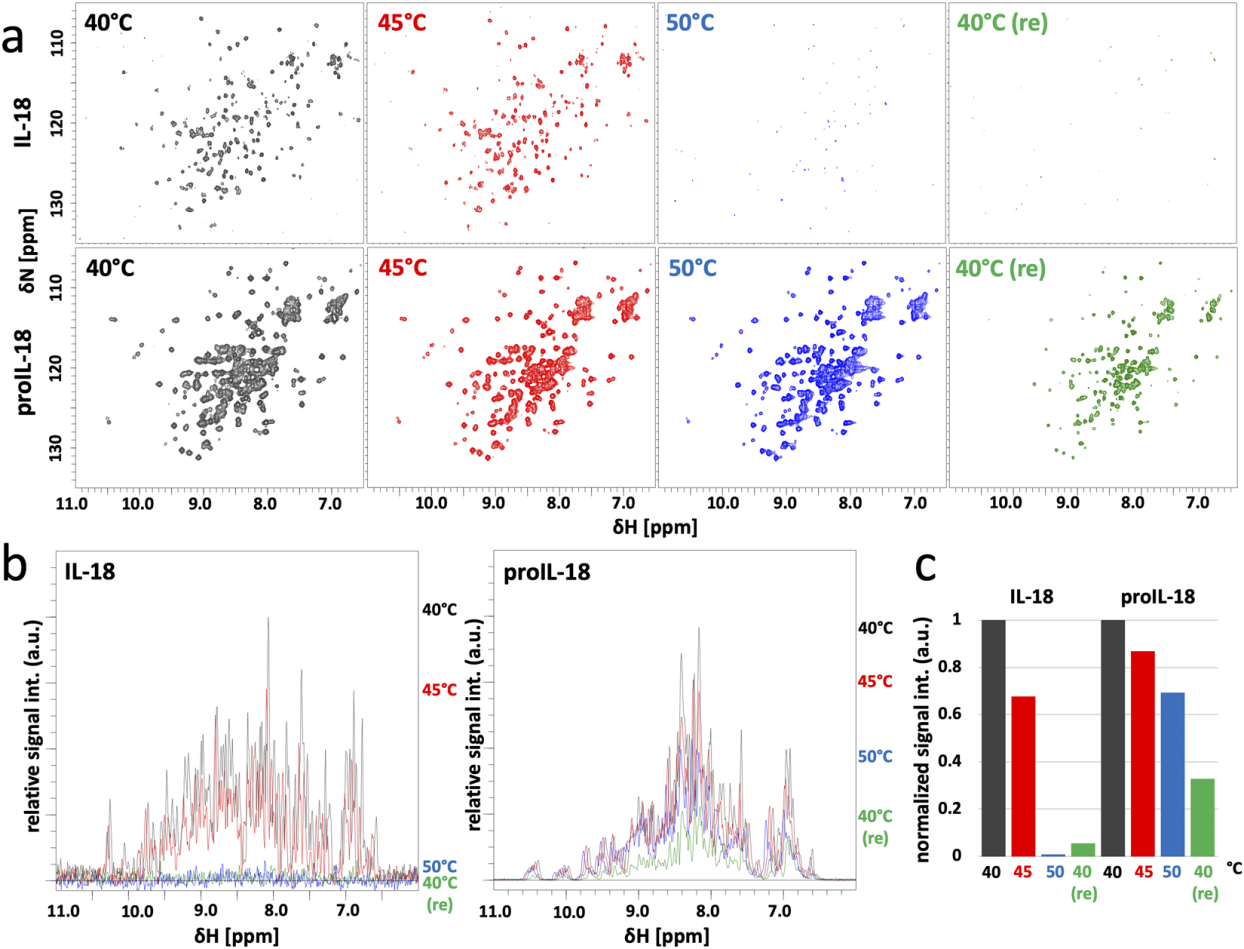
Temperature dependent spectral changes of IL-18 and proIL-18. (**a**) ^1^H-^15^N SOFAST HMQC spectra of IL-18 and proIL-18 (40 °C, black; 45 °C, red; 50 °C, blue; 40 °C (Re), 40 °C after 50 °C measurement and room temperature incubation, green). (**b**) Overlaid ^1^H 1D projections of the IL-18 and proIL-18 spectra in (**a**). (**c**) Changes in the relative peak intensities of (**b**). The peak areas were integrated from 11.0 ppm to 6.0 ppm, and each value was normalized to one at 40 °C. All spectra were measured on a Bruker Avance III 500 MHz in NMR buffer.

The same trend was observed in the high-temperature NMR experiments (Fig. 5a,b). IL-18 formed an irreversible white precipitate at 50 °C after the course of 73 min of measurement, and all cross-peaks disappeared from the spectrum. In contrast, the spectral pattern of proIL-18 was essentially the same between 40 °C and 50 °C, and the signal intensity only decreased by 30% (Fig. 5c). The T_m_ was higher than 50 °C for IL-18 in the temperature scan with the CD measurement (Fig. 4b), whereas the protein precipitated at 50 °C in the NMR study. The discrepancy between these two experiments could be attributable to the difference in the experimental time scales; the temperature in the CD measurements was too fast to allow establishment of a thermal equilibrium state at each temperature. Nevertheless, in both cases, proIL-18 exhibited higher thermostability than IL-18, marking a sharp contrast to proIL-1β, which is substantially less stable than its mature form^53^.

## Discussion

Previous structural studies have revealed that mature IL-18’s N-terminal tyrosine residue is relatively proximal, in the binary complex, to the glycan chain on Asn297 of the primary receptor IL-18Rα^9,54^. Hence, it was likely that the 36-amino acid PP of proIL-18 would sterically hinder binding and so the formation of the signaling complex. In this study, we provided a more detailed structural view of proIL-18 by demonstrating that the PP region extensively interacts with the mature region of proIL-18. Chemical shifts of amide ^1^H suggest that at least a part of the PP region likely adopts a β-strand configuration in proIL-18, implying β-sheet formation between the PP and mature regions. Nevertheless, the resemblance of the CD spectra indicated similar secondary structure content between proIL-18 and IL-18 except for random coils. Thus, a part of the mature region’s β-trefoil structure might have been distorted in proIL-18. The extensively interacting PP region may add additional complexity to determining IL-18’s bioactivities. Due to its potent proinflammatory activity, the IL-18 activity must be tightly regulated. In fact, under normal conditions, blood IL-18 is bound to natural inhibitors, the IL-18 binding protein (IL-18BP)^30^ and soluble IL-18Rs (sIL-18Rα and sIL-18Rβ)^55^, which neutralize its activity. However, in some detrimental autoimmunity or autoinflammatory conditions^18–26^, systemic IL-18 levels are elevated and far exceed those of their antagonists, resulting in the appearance of the active cytokine. Similar tight regulation has also been observed in the IL-1β system, where soluble IL-1 receptors (sIL-1RI and sIL-1RII), a decoy cell-surface receptor (IL-1RII) and an IL-1 receptor antagonist (IL-1Ra) play anti-inflammatory roles *in vivo*. Previous structural characterization of proIL-1β suggested that its PP region prevents the completion of β-trefoil barrel folding by directly interfering with the mature region, although the PP largely adopts a random coil conformation^53^, and thus binding of immature IL-1β to its specific receptors is abrogated. Therefore, PP regions might share a common mechanism for securely suppressing IL-1β/IL-18’s proinflammatory activities.

An intriguing difference between proIL-18 and proIL-1β concerns their folding stability. Our CD and NMR studies have revealed that proIL-18 has an enhanced thermostability relative to the mature protein, which may be advantageous in preserving the precursor in the cytosol for longer durations. This is in sharp contrast to proIL-1β, whose PP in the immature form has been proposed to prevent full barrel formation of the mature region, destabilizing the protein^53^. This difference distinguishes two closely related members of IL-1F, in which IL-18 mRNA and protein are constitutively expressed and stored in the cytosol, whereas IL-1β is transcribed and produced on-demand by various stimuli, such as lipopolysaccharides, and processed to the mature form immediately^56^. IL-1β’s instability may be related to its secretion pathway, where an unconventional autophagy-mediated mechanism has been proposed to be responsible for IL-1β release. In this pathway, less stable proIL-1β is more susceptible to protease-dependent degradation and is, thus, prevented from being secreted^6^. ProIL-18’s higher stability implies that a similar pathway is not used for IL-18 secretion.

The present NMR study demonstrates that proIL-18PP binds to IL-18 to produce dramatic spectral changes in IL-18 (Figs 1–3) that are comparably extensive to those observed when [^15^N]-IL-18 binds the extracellular region of IL-18Rα^9^. Because IL-18BP, a potent IL-18 inhibitor, only mimics one of the three subdomains of IL-18Rα when binding IL-18, it is possible that proIL-18PP interferes with the binding between IL-18 and IL-18Rα. However, the intermolecular binding between proIL-18PP and IL-18 has a lower affinity (tens to 100 μM) than the intramolecular binding between the pro and mature region of proIL-18 because there was no peptide bond between them. Thus, their interaction should be short-lived. Furthermore, peptides are generally susceptible to protease digestion in cell culture and *in vivo*. Hence, proIL-18PP’s inhibitory effect on IL-18 activities may not be very strong. Nevertheless, optimizing its binding properties by changing its amino acid sequence or reformatting it into peptibodies^57^ containing multiple proIL-18PP-like sequences may produce more potent inhibitory compounds. Because proIL-18PP is an endogenous polypeptide generated from proIL-18 upon maturation, its immunogenicity and toxicity should be low if such modifications were minimal; this would be highly beneficial for clinical application, and so proIL-18PP may represent an excellent seed compound to develop IL-18-neutralizing drugs in the future. In this context, our extensive NMR analysis has provided valuable information, i.e., that the central three amino acid residues in proIL-18PP play a key role in mediating its interaction with mature IL-18.

IL-18’s natural inhibitory mechanism could be applied to other IL-1F ligands, which will affect our understanding of IL-1F biology and the development of therapeutics for IL-1F-related diseases.

## Supplementary information

Supplementary Information

## Data Availability

All the unique materials and relevant raw data in this study are available from the corresponding authors upon reasonable request.
