# Supplementary material for: An innate interaction between IL-18 and the propeptide that inactivates its precursor form
Source: Sci Rep. 2019 Apr 16;9:6160. doi: 10.1038/s41598-019-42661-5 (PMC6467916; doi:10.1038/s41598-019-42661-5)
Supplement: Supplementary file 1 — Supplementary Information [file 41598_2019_42661_MOESM1_ESM.pdf]

# Supplementary Information

# An innate interaction between IL-18 and the propeptide that inactivates its precursor form

Naotaka Tsutsumi, Ayumi Yokota, Takeshi Kimura, Zenichiro Kato,
Toshiyuki Fukao, Masahiro Shirakawa, Hidenori Ohnishi (ohnishih@gifu-u.ac.jp)
and Hidehito Tochio (tochio@mb.biophys.kyoto-u.ac.jp)

Scientific Reports (2019)

## SUPPLEMENTARY FIGURES

a

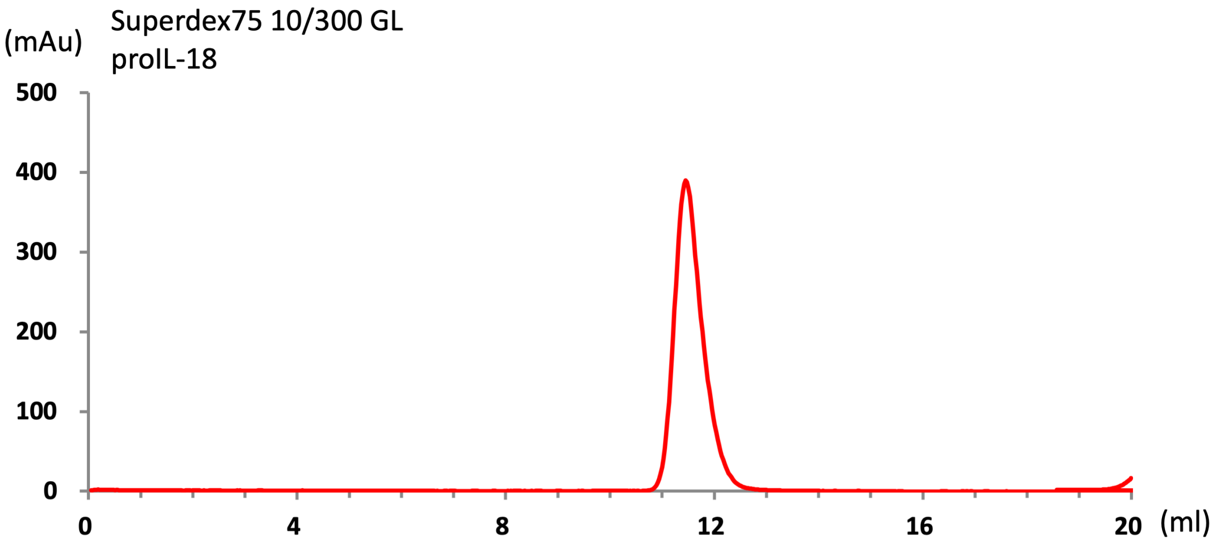


b

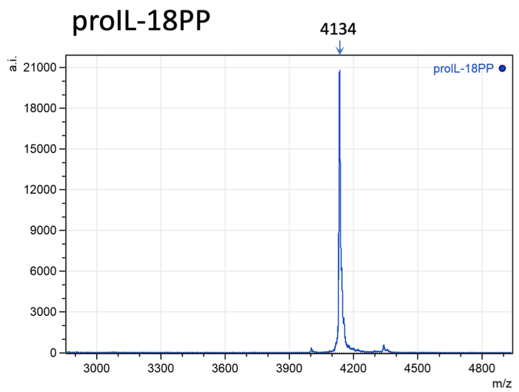


Supplementary Figure 1

Purification of proIL-18's full-length protein and propeptide. (a) Size-exclusion chromatography profile of proIL-18. (b) MALDI-TOF MS spectrum of proIL-18PP.

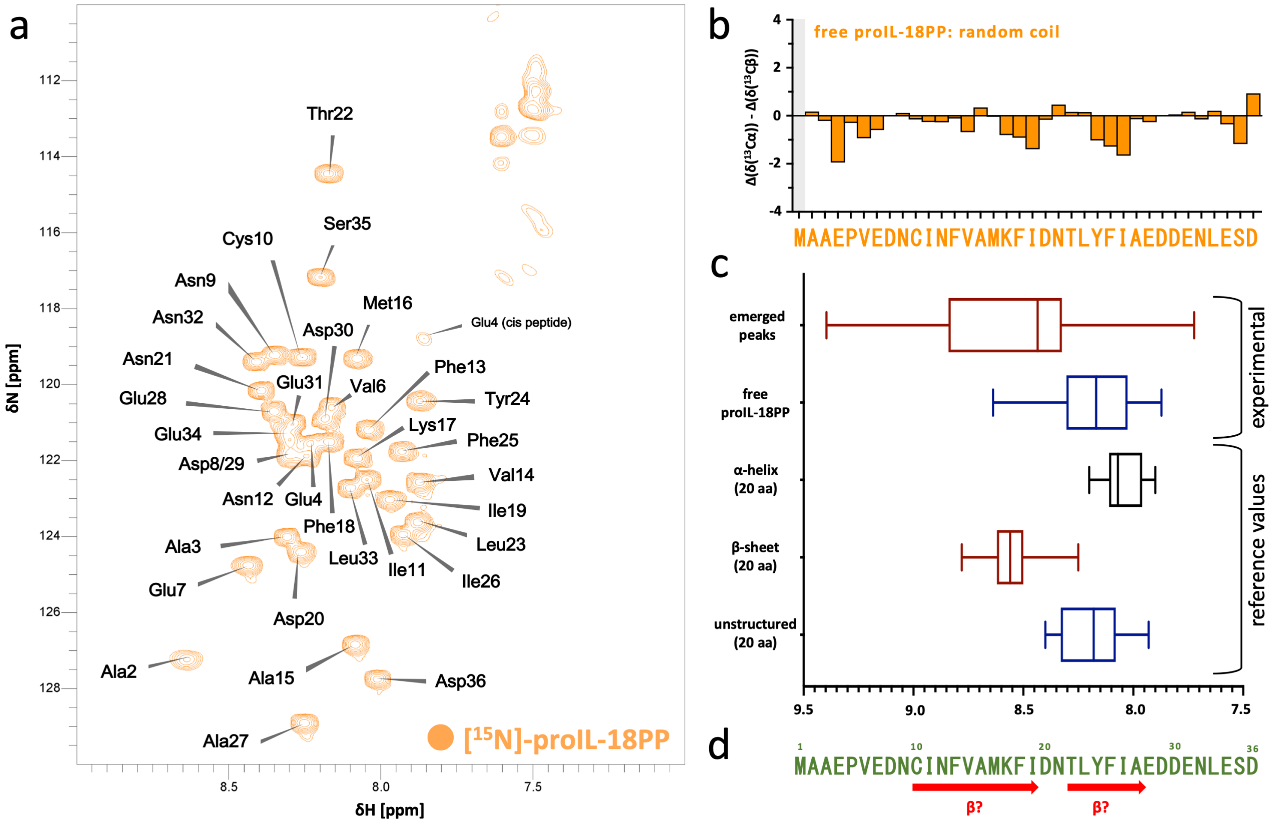


Supplementary Figure 2

Potential secondary structures of proIL-18PP in its free and IL-18-bound states. (a) $^{1}$H-$^{15}$N SOFAST HMQC spectrum of proIL-18PP (residues 1–36) with chemical shift assignments. The spectrum was measured on a Bruker Avance 600 MHz at 25 °C in NMR buffer, whereas the 3D HNCACB/CBCA(CO)NH data set was collected on a Bruker Avance II 700 MHz under the same conditions. The peptide was stable, and there was no observable degradation during measurement. (b) Δ(δ($^{13}$Cα)) - Δ(δ($^{13}$Cβ)) plot of free proIL-18PP, where Δ(δ($^{13}$Cα)) and Δ(δ($^{13}$Cβ)) are the differences between experimental chemical shifts and the corresponding random-coil chemical shifts of reference values for each residue. No secondary structure was detected with TALOS+ (https://spin.niddk.nih.gov/NMRPipe/talos/)[1] using chemical shifts of $^{13}$Cα, $^{13}$Cβ and amide $^{1}$H and $^{15}$N. (c) Box and whisker plots for δ($^{1}$H) of $^{1}$H-$^{15}$N amide cross-peaks. From top to bottom: newly emerged proIL-18PP cross-peaks, free proIL-18PP, average reference values for each of 20 amino acid (aa) residues forming α-helices, β-sheets and random coils[2]. (d) Secondary structure prediction of proIL-18PP prepared using PSI-blast based secondary structure PREDiction (PSIPRED) v4.0 (bioinf.cs.ucl.ac.uk/psipred_new/)[3].

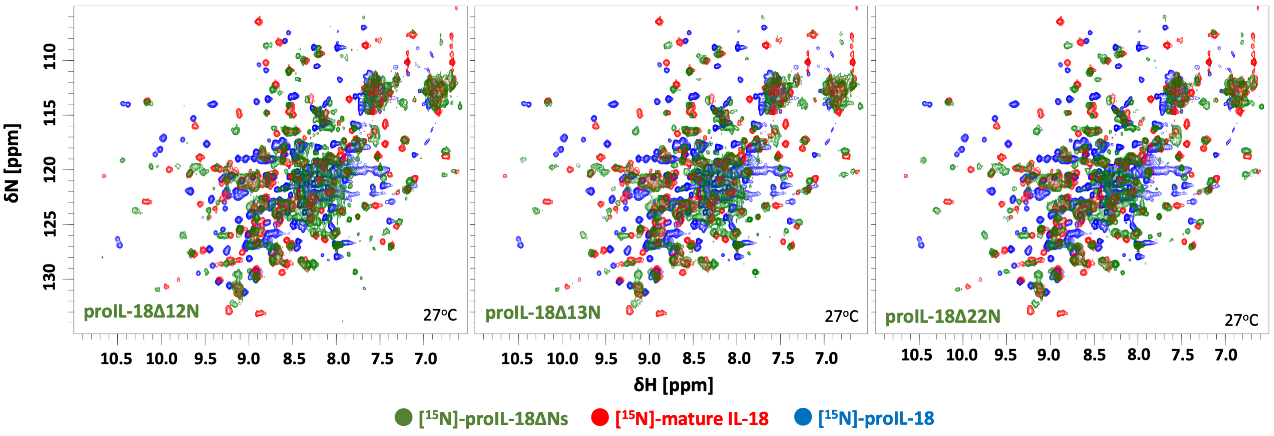


Supplementary Figure 3

$^{1}$H-$^{15}$N correlation NMR spectral comparison of [$^{15}$N]-proIL-18Δ12N, [$^{15}$N]-proIL-18Δ13N and [$^{15}$N]-proIL-18Δ22N with proIL-18 and IL-18. All three proIL-18ΔN variants showed essentially the same spectra and were more similar to IL-18 than proIL-18.

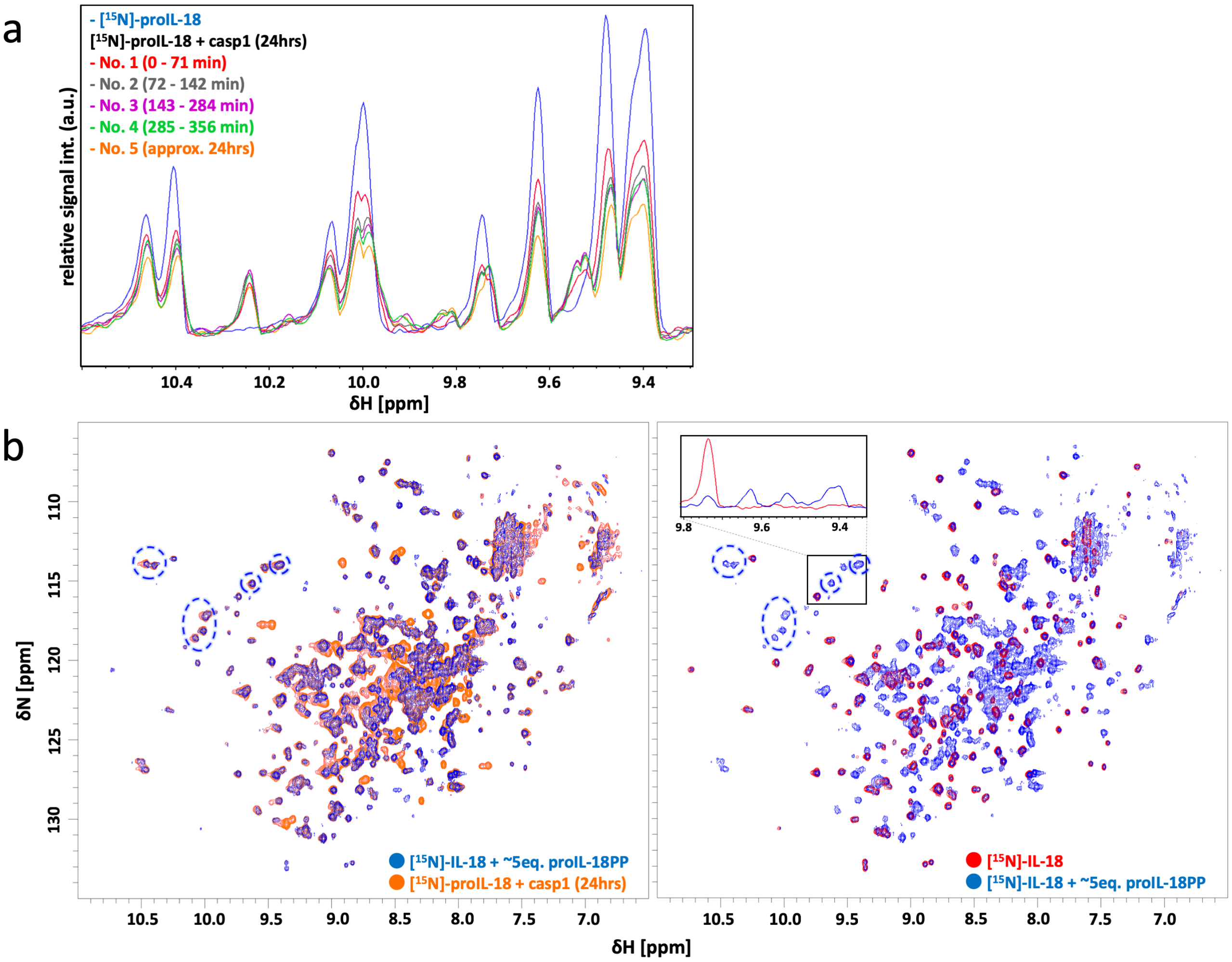


Supplementary Figure 4

(a) Overlaid 1D $^1$H projections for the six $^1$H-$^{15}$N spectra of [$^{15}$N]-proIL-18 during caspase-1 digestion. The time points from the start of the reaction are indicated in the figure, and projections are prepared using the square region in Fig. 3b. (b) Left: Spectral comparison of [$^{15}$N]-IL-18 in the presence of approx. 5eq. of proIL-18PP (blue, the same spectrum as Fig.3e pink) and caspase-1-cleaved [$^{15}$N]-proIL-18 (orange, from Fig. 3b). Right: Overlaid spectra of [$^{15}$N]-IL-18 in the absence (red, from Fig. 3d) and presence (blue, from left) of non-labeled proIL-18PP. Representative signatures from [$^{15}$N]-proIL-18 spectra are marked by blue dashed circles. The squared region in the right spectra was selected to be displayed as 1D $^1$H projections, to show examples of the peak decay and emergence during the titration. All $^1$H-$^{15}$N SOFAST HMQC spectra were measured on a Bruker Avance II 700 MHz at 37 °C in caspase-1 reaction buffer.

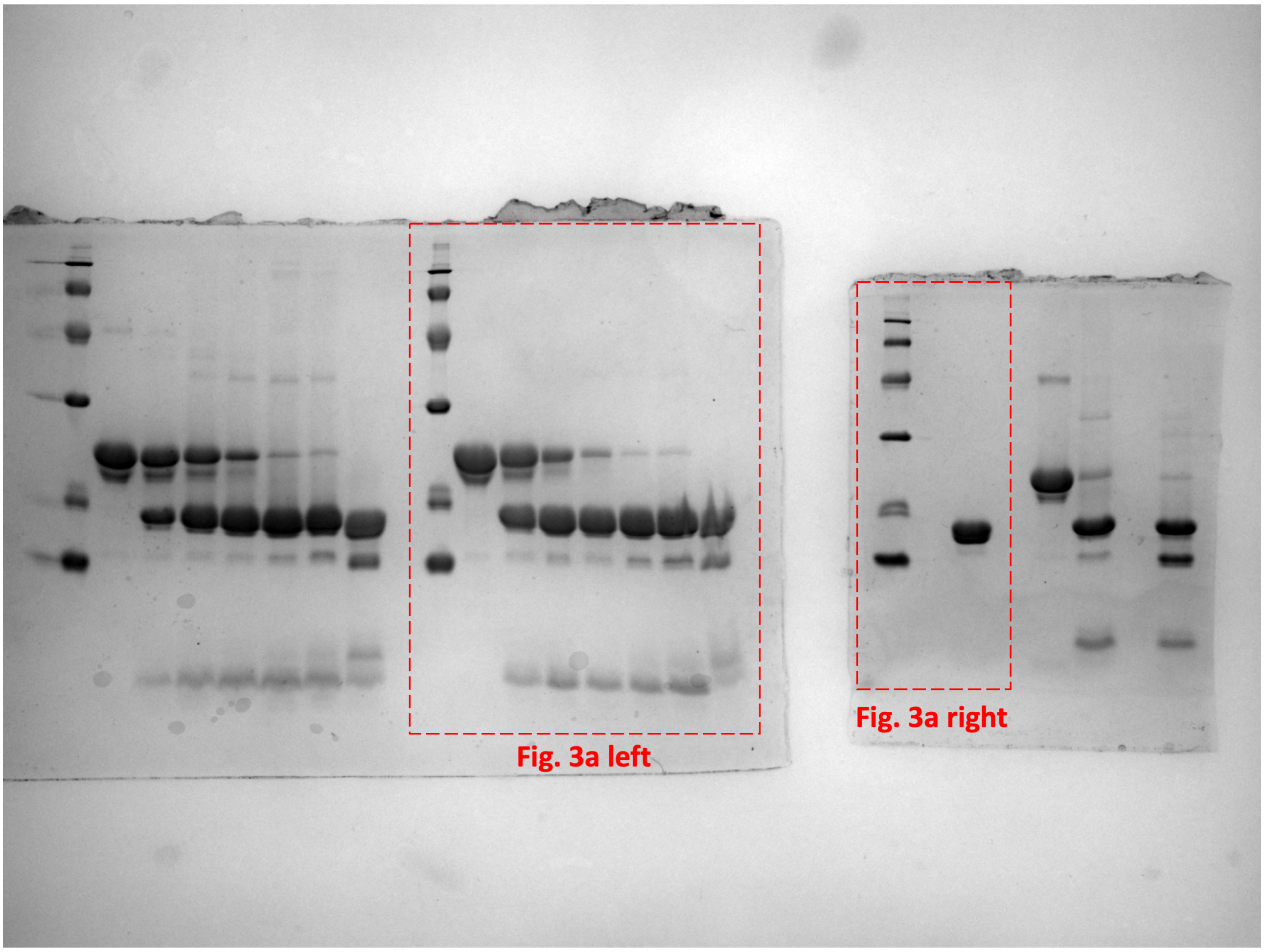


Supplementary Figure 5

Uncropped image of SDS-PAGEs used to prepare Fig. 3a.

## SUPPLEMENTARY REFERENCES

1. Shen, Y., Delaglio, F., Cornilescu, G. & Bax, A. TALOS+: A hybrid method for predicting protein backbone torsion angles from NMR chemical shifts. *J. Biomol. NMR* **44**, 213–223 (2009).
2. Wishart, D. S., Sykes, B. D. & Richards, F. M. Relationship between nuclear magnetic resonance chemical shift and protein secondary structure. *J. Mol. Biol.* **222**, 311–333 (1991).
3. Buchan, D. W. A., Minneci, F., Nugent, T. C. O., Bryson, K. & Jones, D. T. Scalable web services for the PSIPRED Protein Analysis Workbench. *Nucleic Acids Res.* **41**, 349–357 (2013).
